# Evaluation of Dentaport ZX and Raypex 6 electronic apex locators: An in vivo study

**DOI:** 10.4317/medoral.19114

**Published:** 2013-10-13

**Authors:** Saddy Moscoso, Kenneth Pineda, Juan Basilio, Carlos Alvarado, Miguel Roig, Fernando Duran-Sindreu

**Affiliations:** 1 MD, Department of Restorative Dentistry and Endodontics, Universitat Internacional de Catalunya, Barcelona, Spain; 2 MD, DDS, PhD, Department of Restorative Dentistry and Endodontics, Universitat Internacional de Catalunya, Barcelona, Spain; 3 Department of Restorative Dentistry and Endodontics, Universitat Internacional de Catalunya, Barcelona, Spain

## Abstract

Introduction: Raypex 6 is an electronic apex locator (EAL) that has not yet been tested in vivo. The purpose of this in vivo study was to compare the accuracy of two EALs: the Dentaport ZX and the Raypex 6. Material and Methods: The study involved 36 straight single-rooted teeth. A 10-K file was advanced until the EAL detected the major foramen. The file was fixed in a replaceable pattern of light-cured composite. The apical part of each canal was trimmed to expose the file tip. The distances from the file tips to the major foramen were measured. Results: Wilcoxon’s signed Rank test found no significant differences between the Dentaport ZX and Raypex 6 in terms of their abilities to detect the major foramen (P = .52) The Dentaport ZX was accurate 82.35% of the time to ± 0.5 mm and 97.05% of the time to ± 1 mm, whereas the Raypex 6 was accurate 88.22% of the time to ± 0.5 mm and 100% of the time to ± 1 mm. Conclusions: No statistically significant differences were observed between the performance of the Dentaport ZX and Raypex 6 EALs under the in vivo clinical conditions used in this study.

** Key words:**Dentaport ZX, electronic apex locator, major foramen, Raypex 6, working length.

## Introduction

Working length (WL) is defined as “the distance from a coronal reference point to the point at which canal preparation and obturation should terminate” (1). The correct determination of the WL is a key factor for successful root canal treatment, because it reduces the possibility of insufficient debridement of the canal or damage to the periapical tissues due to over-instrumentation (2-4). Traditionally, the WLs of root canals have been determined by the interpretation of a radiograph of an instrument placed in the canal. However, the radiographic evaluation of the WL may prove inaccurate, depending on the direction and extent of the root curvature and the relationship between the major foramen and the anatomic apex (5-7). In addition, because the radiographic image obtained is two-dimensional, and the tooth is three-dimensional, distortions of the radiographs may cause miscalculation of the WL. Electronic apex locators (EALs) can determine the WL more accurately than radiographic methods (8).

Whereas first-generation EALs measured resistance, second-generation EALs measured impedance. The main drawback of both types of EAL was the poor accuracy caused by electrolytes. This limitation was overcome with the introduction of third-generation EALs, such as the Root ZX (J Morita Corp, Tokyo, Japan). The Root ZX uses the ‘‘ratio’’ method to measure the root canal length. This method involves the simultaneous measurement of impedance at two frequencies (0.4 kHz and 8 kHz), and calculation of a quotient that expresses the position of the file tip in the canal (9). The Root ZX apex locator is considered to be the gold standard against which newer EALs are evaluated (10). Many studies have addressed the benefits and clinical performances of the diverse range of EAL models that have been developed in recent years. However, manufacturers make contrasting claims for their products, so it remains difficult for the practitioner to choose from the EALs available.

To the best of our knowledge, no in vivo studies have evaluated the accuracy of the recently developed Raypex 6. Given the absence of reports related to the accuracy of the Raypex 6, the purpose of the study reported herein was to compare the accuracy of the Dentaport ZX, the latest version of the Root ZX, and the Raypex 6 in detecting the major foramen.

## Material and Methods

We selected 36 maxillary and mandibular single-rooted teeth with mature apices in 15 patients. The teeth were scheduled for extraction for periodontal, prosthetic, or orthodontic purposes. Teeth with metallic restorations, fractures, root resorption, or open apices were not included. All of the teeth responded positively to tests for sensitivity to cold, and clinically, all the pulps were confirmed to be vital on examination of their chambers. Informed consent was obtained from each patient in accordance with approval of the study by the Ethical Board of the Universidad de San Carlos de Guatemala. All clinical procedures and measurements were conducted by a single operator.

The teeth were isolated using a rubber dam under local anesthesia. Endodontic access was performed, and the coronal portion of each canal was flared using an SX Protaper file (Maillefer, Ballaigues, Switzerland). Each canal was then irrigated with 4% NaOCl. Excess fluid was removed from the pulp chamber using an air syringe, but no attempt was made to dry the canals.

For both of the Raypex 6 and Root ZX devices, the clip was attached to the patient’s lip, and the electrode was connected to a 10-K file. With the Raypex 6, the file was advanced within the root canal until the red bar began flashing, which indicates the major foramen (according to the manufacturer’s instructions). Using the Dentaport ZX, the file was advanced until the “APEX” signal appeared on the liquid crystal display. Measurements were considered to be valid if they remained stable for at least 5 seconds. For the first EAL used, the 10-K file was fixed at the WL that had been determined electronically with a removable flowable light-cured composite (3M Espe, St. Paul, MN, USA). Following this, the composite pattern containing the file was withdrawn from the tooth. In each case, it was important that the composite pattern could be repositioned in the same location over the respective tooth. The WL was rechecked electronically after the K file had been fixed to confirm that it was in the correct position. The procedure was then repeated for the same tooth with the second EAL. Because both EALs were used in each canal, the first EAL to be used was alternated for each successive tooth. The apical portion of the root was trimmed longitudinally using a fine diamond bur (Gebr. Brasseler, Lemgo, Germany) while being visualized using a microscope (D.F. Vasconcellos, Sao Paulo, Brazil) at ×16 magnification to expose the file tip. The additional tooth structure was removed carefully using an OptiDisc Coarse/Medium Soflex Disc (KerrHawe, Bioggio, Switzerland) until the file tip and the root canal were visible. The apical parts of the specimens were photographed twice (once for each EAL) using a digital camera (Ken-A-Vision, Kansas City, MO, USA) after visualization using a stereomicroscope (Meiji Techno, Saitama, Japan) at ×30 magnification. The first and second photographs were taken with the composite pattern repositioned for measurement with the Dentaport ZX and the Raypex 6, respectively.

Two teeth were excluded from the study because they fractured during extraction. Hence, 34 teeth were included in the statistical analysis. Two operators together marked the major foramen (the most coronal border of the major foramen). The distance between the major foramen and the file tip was measured on each image, and recorded as either negative (if the file tip was short of the major foramen), or positive (if the file tip was beyond the major foramen). Measurements of the WL were compared between the EALs using the Wilcoxon’s signed rank test for nonparametric data because the data for the Dentaport ZX group was not normally distributed (goodness-of-fit Shapiro–Wilks test = 0.001). Statistical significance was set at P < .05.

## Results

The Wilcoxon’s signed rank test failed to show a significant difference (P > .05) when comparing the median differences between the abilities of the Dentaport ZX and Raypex 6 to detect the major foramen (P = .52). The Dentaport ZX was accurate 82.35% of the time to ± 0.5 mm and 97.05% of the time to ± 1 mm, whereas the Raypex 6 was accurate 88.22% of the time to ± 0.5 mm and 100% of the time to ± 1 mm ( Table 1).

**Table 1:**
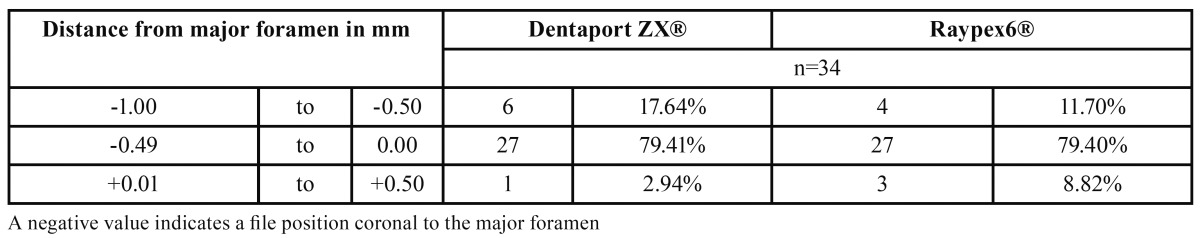
Position of the file tip relative to the major foramen as determined by Dentaport Zx and Raypex 6.

## Discussion

The apical constriction is the narrowest part of the root canal, and is regarded as the physiological apical limit for instrumentation and filling of the root canal system (2-3). However, there are problems with adopting this particular limit. For example, the existence of an apical constriction might be more conceptual than real because fewer than half of the teeth examined have a traditionally conceptualized single apical constriction (11). Moreover, several researchers have suggested that the precise location of the AC cannot be determined (12-13), and there might not always be an apical constriction (12-13). Consequently, we and many others have evaluated the accuracy of EALs in determining the WL, taking as a reference point the major foramen or a point 0.5 mm short of the major foramen (14-17).

Inconsistent measurements in laboratory studies that have evaluated EALs may be explained by procedural errors, by bias that results from the inaccurate adjustment of the stopper to the reference point, or by movement of the stopper during the measurement procedure (18-19). In our study, the file was cemented in position using a flowable light-cured composite resin. Furthermore, the distance between the instrument and the major foramen was measured after longitudinal wear that had been performed on the apical portion of the root (14-17,19,20), because it reduces the number of variables involved and enables a more accurate calculation of the distance between the file and the major foramen (19).

Precise comparison of the accuracy of different types of EAL in determining the WL is possible only if the same teeth are assessed by all the devices. Therefore, we followed the protocol used by Wrbas et al. (20), which allowed us to calculate the accuracy of the two EALs in the same teeth.

When detecting the major foramen, the Dentaport ZX was accurate 82.35% of the time to ± 0.5 mm and 97.05% of the time to ± 1 mm. The findings of the present study are similar to those obtained previously. D’Assunção et al. (21), Fellipe et al. (22), and Pagavino et al. (23) reported that the Root ZX was accurate in determining the major foramen to ± 0.5 mm in 89.7%, 86%, and 82.75% of the samples, respectively. Given that no studies appear to have evaluated the accuracy of the Raypex 6 EAL, it was not possible to compare our results obtained using the Raypex 6 with those from previous studies. In our study, the Wilcoxon’s signed rank test failed to show a significant difference when comparing the median differences between the abilities of the Dentaport ZX and Raypex 6 to detect the major foramen. In the present study, the mean distance from the major foramen to the file tip measured –0.25 ± 0.26 mm for the Dentaport ZX and –0.26 ± 0.24 mm for the Raypex 6.

The Root ZX shows a strong tendency to overestimate the WL (8,23,24). These results differ from those obtained in the present study and other studies in which many more short measurements were made than long measurements (21,22). The different results obtained among these studies might be explained, at least in part, by the nature of the teeth examined, because the diameter of the minor and major foramen and the location of the major foramen are three important factors that affect the performance of EALs (23,25,26).

## Conclusions

No statistically significant differences were observed between the Dentaport ZX and Raypex 6 EALs under the in vivo clinical conditions used in this study.
